# Women’s cancers: how the discovery of BRCA genes is driving current concepts of cancer biology and therapeutics

**DOI:** 10.3332/ecancer.2019.904

**Published:** 2019-02-14

**Authors:** Pooja Murthy, Franco Muggia

**Affiliations:** 1 New York University School of Medicine, New York, NY 10016, USA; 2 Maimonides Cancer Center, Brooklyn, NY 11220, USA

**Keywords:** ovarian cancer, breast cancer, BRCA, BRCA1, BRCA2, homologous recombination, DNA repair, DNA damage response, high-grade serous ovarian cancer, triple negative breast cancer, PARP inhibitor, synthetic lethality, bilateral salpingo-oophorectomy, prophylactic mastectomy, chemoprevention, immunotherapy

## Abstract

Over the last two decades, discoveries related to the breast cancer susceptibility genes 1 and 2 (*BRCA1* and *BRCA2*) have profoundly changed our understanding and management of hereditary breast and ovarian cancers. The concept of synthetic lethality, which arises when cells become vulnerable to a combination of deficiencies in DNA repair, has driven the expanding roles of poly (adenosine diphosphate (ADP)-ribose) polymerase inhibitors in breast and ovarian cancers, and prevention strategies are taking into account the tissue specificity, natural history (fallopian tube origin of some high-grade serous ovarian cancers) and hormone sensitivity of BRCA-associated cancers. Current research has focussed on further elucidating the roles of BRCA proteins in DNA repair, investigating other key DNA repair processes and proteins and linking aberrant DNA repair with carcinogenesis. The ultimate goal is to translate this evolving knowledge into improving the clinical care and treatment of patients with pathogenic *BRCA* variants or other deficiencies in homologous recombination (HR). In this review, we will discuss 1) the role of BRCA proteins in DNA repair; 2) emerging concepts in the biology of HR deficiency and 3) implications for prevention and treatment.

## Introduction and historical landmarks

Family studies first by Henry Lynch and then by others pointed to a genetic origin for familial breast and ovarian cancers, with lifetime risks exceeding 50% in such family members. Interventions to diminish these risks by prophylactic surgeries or by drugs aiming to diminish hormonal actions on the breast (using surrogate endpoints such as breast density) were introduced once the hereditary nature of this predisposition was defined. Mary-Claire King’s genetic studies indicated a locus on chromosome 17p for a putative susceptibility gene. Shortly thereafter, identification and cloning of the breast cancer susceptibility genes 1 and 2 (*BRCA1* and *BRCA2*) [1, 2] took place and led to a marked expansion of our knowledge on genetic susceptibility, its function and impact on tumour biology, and both preventive and therapeutic interventions. Soon after the cloning of the *BRCA* genes, Livingston’s laboratory and others found that loss of BRCA function was associated with a defect in homologous recombination (HR), a critical DNA repair pathway [3, 4]. Mouse models replicated the consequences of silencing *BRCA* genes in the development of triple negative breast cancer and demonstrated the effectiveness of DNA damaging agents such as platinum agents in their treatment. More recently, these studies have given way to the concept of synthetic lethality, which arises when cells become vulnerable to a combination of deficiencies in DNA repair, whereas a deficiency in only one pathway may not be lethal, thereby allowing for tumour-specific toxicity. This concept drove the initial development of poly (ADP-ribose) polymerase (PARP) inhibitors in the seminal phase I study of the PARP inhibitor olaparib in patients with advanced ovarian cancer and germline *BRCA* mutations [5]. The development of additional agents targeting PARP in other oncologic areas, both as single agents and in combination with other drugs, stalled for some time but eventually culminated in the 2014 Food and Drug Administration (FDA) approval of olaparib for the treatment of patients with deleterious or suspected deleterious germline *BRCA*-mutated advanced ovarian cancer who had progressed on three or more lines of chemotherapy [Lynparza prescribing information, 2014].

Table 1 features the highlights of conferences relevant to BRCA biology over the past 15 years.

## Cancer risk overview and epidemiology

Hereditary breast and ovarian cancers are primarily related to highly penetrant germline mutations in either one of the two breast cancer susceptibility genes, *BRCA1* and *BRCA2* [6–8]. Carriers are heterozygous in one germline allele, and cancer may develop with loss of the wild type allele. Among women with ovarian cancer, regardless of family history, approximately 15% carry germline *BRCA* mutations [9]. In the general population of women with breast cancer in Western countries, 4%–5% carry deleterious *BRCA* mutations [10, 11], increasing to 12% in women who are less than 40 years old at the time of diagnosis [12]. Prevalence rates are also high among certain ethnicities; 10%–12% of breast cancers in the Ashkenazi Jewish population, unselected for family history, are attributable to mutations in *BRCA1* or *BRCA2* [13].

The cancer risks for patients with one of the three germline founder mutations in *BRCA1* and *BRCA2* have been extensively described. Similar information has gradually emerged for *BRCA1* and *BRCA2* variants across various ethnicities. One prospective cohort study evaluating over 9,000 mutation carriers, the majority from Europe, found the cumulative breast cancer risk to age 80 years was 72% for *BRCA1* and 69% for *BRCA2* carriers. The cumulative ovarian cancer risk to age 80 years was 44% for *BRCA1* and 17% for *BRCA2* carriers [14]. The overall risk of pancreatic cancer is about 1% and 4.9% for *BRCA1* and *BRCA2* mutation carriers, respectively [15, 16]. The prostate cancer risk is also increased and may range from 9% in *BRCA1* mutation carriers to 33% in *BRCA2* mutation carriers [17, 18]. Risks for melanoma, skin cancer, other gastrointestinal cancers and endometrial cancer may also be increased, but are not well characterised, and are often found with advancing age in individuals who have been successfully treated earlier for either breast or ovarian cancer [18]. Less is known about cancer risks for rarer mutations such as PALB2 and other genes that relate to the HR pathway.

Much remains to be learned about the prevalence of pathogenic *BRCA* variants in unselected patients with breast and ovarian cancers. The initially noted high penetrance of *BRCA* mutations has contributed to the ongoing interest in studying the specific pathogenesis, management and treatment issues for this subset of patients.

## Overview of DNA repair and roles of BRCA and related genes

### Repair of DNA damage

DNA repair processes are essential in maintaining genetic integrity. The repair of a double-stranded DNA break (DSB) is particularly critical since an unresolved DSB often leads to genetic instability and cell death [19]. DSBs can be repaired through one of four possible mechanisms: nonhomologous end joining (NHEJ), HR, single-strand annealing (SSA) and microhomology-mediated end joining (MMEJ).

In NHEJ, the DSB is joined through blunt end ligation [20, 21]. Although this pathway has the potential to restore the original DNA sequence, any processing of the ends prior to ligation through nucleases or polymerases can lead to deletion or insertion mutations [22]. NHEJ is consequently error-prone but is evolutionarily conserved perhaps due to its fast kinetics, relative independence from the cell cycle and mechanistic flexibility [21].

Instead of NHEJ, the DSB can be initially resected, leaving two 3’ single-stranded overhanging ends. Then, the break can be repaired through one of three possible mechanisms: SSA, MMEJ and HR. SSA relies on the presence of a homologous sequence on either side of the break. After the creation of the 3’ overhanging ends, these homologous sequences anneal to each other and the intervening sequences are deleted. The break is thus repaired but there is a loss of genetic information [23]. MMEJ is an error-prone DSB repair mechanism that uses short homologous sequences (microhomology) flanking the DSB to align the ends for repair and is associated with deletions, insertions and chromosomal translocations [24]. Some of the distinctions between SSA and MMEJ include different mediators of synapsis and differences in the length of the annealing intermediate (i.e. MMEJ uses very short homologous sequences whereas SSA uses longer stretches of homology) [25].

HR is the DSB repair process in which one of the strands from the DSB invades into a homologous template, preferentially the identical sister chromatid [26], and uses the homologous sequence as a template for nascent DNA. Since HR allows for faithful replication and repair based on the homologous sequence, the process generally preserves the reading frame, and in most cases, is error-free. This is in contrast to the error-prone NHEJ, SSA and MMEJ pathways.

The question regarding how a cell ‘chooses’ between the different DSB repair pathways in a specific biological context is beyond the scope of this review. The decision is certainly multifactorial, but many of the details have yet to emerge. Briefly, the stage of the cell cycle, availability of the sister chromatid, the balance of mediators of synapsis (including BRCA 1 and 2, RAD51, PARP, DNA polymerase theta, REV7, 53BP1, ataxia telangiectasia mutated (ATM) and many other key proteins), the chromatin context adjacent to the break, the nature of the break itself and tissue-specific factors all likely play a significant role [25, 27–30].

### BRCA proteins in homologous recombination

HR is distinguished by its error-free repair of DSBs and is the predominant and promoted DSB repair process in the S/G2 phases of the cell cycle. The steps of HR have been reviewed in other articles [31–33], but we will present a brief, somewhat simplified overview of HR in mammalian cells here, highlighting the roles of BRCA1 and BRCA2.

Initially, ATM and ATM and RAD3-related (ATR) recognise the DSB and phosphorylate downstream targets. BRCA1, along with BARD1 and BRIP1, act as a scaffold to organise other proteins, including the Mre11-Rad50-Nbs1 (MRN) complex and C-terminal binding protein interacting protein (CtIP), which then facilitate end resection [34–36]. Antagonising mediators, such as 53BP1 and RIF1, may inhibit end resection at DSBs [29, 37, 38], shuttling the DSB into the NHEJ repair process instead. The end resection is extensive, in contrast to the less extensive end resection that can lead to the MMEJ pathway. Phosphorylated replication protein A binds to the resected end, protecting it and also facilitating the next step—Rad51 loading [39].

Strand invasion, the defining step of HR, may then occur. BRCA1 promotes the recruitment of BRCA2 to the newly end resected-DSB through the bridging protein PALB2 [40, 41]. BRCA2 then helps load the RAD51 strand-exchange protein complex onto the overhanging 3’ break end. Next, the RAD51 single-stranded-DNA filament invades the homologous DNA strand, which, in most cases, is the sister chromatid. The remainder of the repair occurs with the use of the sister chromatid as the homologous template [31–33].

BRCA2 and BRCA1 also function in another critical process: the protection of stalled replication forks. More specifically, BRCA1 and components of the Fanconi anaemia pathway may cooperate with BRCA2 in preventing nascent DNA degradation at stalled replication forks [42]. Some studies have shown that this process of fork stabilisation may be independent of HR [43]. Broadly speaking, emerging data point to a complex interplay of HR factors in fork remodelling and stability [44].

Additionally, it is important to note that BRCA1 and BRCA2 are involved in processes other than HR and DNA repair; recent studies have found that the proteins play important roles in cell cycle progression, transcriptional control, mammary development and mitosis itself [45, 46].

### Phenotypic expression of homologous recombination deficiency (HRD)

***HRD biomarkers—***Cells become deficient in HR through loss of BRCA function or loss of function of any other protein/factor involved in the process. Assays for HRD have focussed on the detection of underlying driver mutations (ATM, alpha thalassemia/mental retardation syndrome X-linked (ATRX), BARD1, Bloom’s syndrome gene product (BLM), BRIP1, FANCA/C/D2/E/F/G/L, MRE11A, nibrin (NBN), PALB2, phosphatase and tensin homolog (PTEN), RAD50, RAD51, RAD51B and Werner syndrome helicase (WRN)), epigenetic changes such as *BRCA* promoter methylation, or the resultant mutational landscape of deficient HR (copy number alterations, structural rearrangements, including telomeric allelic imbalance, large-scale transition and loss of heterozygosity (LOH)) [47]. Head-to-head comparisons of the different HRD assays have not yet been done, and so it is not clear which assay is superior. However, the two most clinically validated HRD assays are the Myriad Genetics myChoice HRD Plus assay and the Foundation Medicine’s FoundationFocus companion diagnostic (CDx) xBRCA LOH assay. The Myriad myChoice assay measures telomeric allelic imbalance, large-scale transition and LOH. FoundationFocus CDxBRCA LOH is an assay that uses next-generation sequencing for the qualitative detection of *BRCA1* and *BRCA2* sequence alterations and LOH.

A few prospective clinical trials in breast and ovarian cancer have used these assays not only to stratify patients with or without HRD but also as inclusion or exclusion criteria. Although showing that HRD assays may have predictive value for response to platinum chemotherapy and PARP inhibitors, their ultimate role in selecting patients that may benefit from these drugs is unclear [48–51], especially as the indications for PARP inhibitors have broadened beyond patients with germline *BRCA* mutations to include patients with HRD from other causes and even to other ovarian cancers without identifiable defects in DNA repair. Additional data on the clinical use of HRD assays may emerge in the future.

Most of these assays focus on genomic signatures or scars, which may or may not represent the tumor’s current HR capability. Cells with HRD may in time regain their ability to perform HR through reversion mutations or other mechanisms, and therefore, most of the current assays are limited in identifying such tumours since they may be unable to capture these new mutations. Functional assays for HRD should be able to overcome these limitations but remain in the research arena since they require fresh tissue.

***HRD and tissue-specific tumour development—***An unanswered question in *BRCA*-related cancer biology is why HR defects are associated with cancer development both in terms of tissue specificity (breast and ovary) and histology (i.e. high-grade serous predominance).

*BRCA*-mutation-related sex hormone alterations have been sought to explain carcinogenesis in hormone-sensitive tissues such as the breast and ovary. BRCA1 can interact with oestrogen and progesterone receptors to decrease sex hormone transcription [52], and premenopausal women with germline *BRCA* mutations have been found to have higher serum progesterone levels in the luteal phase of the menstrual cycle than non-carriers [53]. Mice models of *BRCA1*-deficient breast tumours have shown increased progesterone receptor expression in both the breast tumours and the adjacent benign tissue [54], and that mammary tumorigenesis can be prevented with progesterone inhibition [55]. The receptor activator of nuclear factor kappa-B ligand (RANKL) is an important paracrine mediator of progesterone signalling in breast tissues. Preclinical studies have shown that RANK-RANKL signalling is augmented in a population of luminal progenitor cells from precancerous breast tissues with heterozygous *BRCA1* mutations, and that pharmacological inhibition of the RANK/RANKL pathway delayed the onset and reduced incidence of breast tumours in mice models [56–58]. Therefore, inhibition of the RANK-RANKL pathway has become an active focus of the clinical investigation on the prevention of *BRCA*-associated breast cancers.

Oestrogen may also play a role; oestrogen can overcome HRD-related oxidative stress-induced cell death through induction of the transcription factor NRF2, which regulates an antioxidant pathway [59, 60]. Oestrogen may act through the PI3K - protein kinase B (PKB, also known as AKT) - mammalian target of rapamycin (mTOR) pathway to induce NRF2 accumulation [59, 61], supporting the emerging clinical strategy of treating *BRCA1*-related cancers with PI3K inhibitors. Targeting the PI3K-AKT-mTOR pathway has established a basis in breast cancer treatment. Everolimus, an mTOR inhibitor, is approved and has demonstrated activity in combination with exemestane in hormone receptor-positive, HER2-negative breast cancer [62]. Most recently, the PI3K inhibitor alpelisib also showed a progression-free survival benefit in hormone receptor-positive, HER2-negative breast cancer [63].

Fallopian tube origin of *BRCA*-mutation driven high-grade serous ovarian cancers may also reflect the contribution of oestrogen-dependent fimbrial proliferation in their pathogenesis, beginning with TP53 immunostaining and the serous tubal intraepithelial cancer (STIC) [64, 65].

***Haploinsufficiency—***Patients with germline *BRCA* mutations are heterozygous at the mutated *BRCA* allele and their heterozygous cells are assumed to be biologically normal until alteration of this unmutated allele occurs, resulting in loss of BRCA functionality. More recently, Pathania et al. [66] showed that human mammary epithelial cells and fibroblasts with heterozygous *BRCA* mutations (*BRCA1^mut/+^*) are intrinsically ‘haploinsufficient’ in BRCA-dependent stalled fork repair when compared to mammary epithelial cells and fibroblasts without *BRCA* mutations (*BRCA1^+/+^*). When subjected to ultraviolet (UV) radiation, heterozygous cells exhibited multiple defects of stalled fork repair resulting in increased replication stress and DNA breaks compared to wild type cells, while retaining proficiency in other BRCA1 functions such as mitosis, cell cycle control, mammary development and heterochromatin-based satellite RNA suppression. HR-mediated repair of double-stranded breaks, while unaltered by infrared radiation, was demonstrably impaired if replication stress was induced via increasing exposure to UV followed by infrared irradiation—as assessed by RAD51 recruitment and olaparib cytotoxicity in comparison to wild type *BRCA* cells. This ‘conditional’ haploinsufficiency in HR may be expected to occur when seemingly normal cells are subjected to a certain threshold of replication stress [66]. Interestingly, Winqvist et al. [67] found that B lymphocytes and T cells from PALB2 heterozygotes were also haploinsufficient for replication stress responsiveness, suggesting that the defect could extend to mutations in other genes associated with BRCA function. At present, one can only speculate that such deficient stalled fork repair may lead to tumorigenesis: could secondary squamous cell carcinomas of the oral cavity in *BRCA*-mutated patients exposed to radiation or certain types of chemotherapy, or skin cancers and melanomas in excessively sun-exposed subjects be manifestations of haploinsufficiency?

## Prevention and treatment of BRCA-mutated ovarian and related gynaecologic cancers: current status

### Prevention

Individuals with a germline pathogenic *BRCA1* or *BRCA2* mutation should be counselled at the time of disclosure of the test results and options for primary prevention should be discussed. Unfortunately, there is a lack of high-quality data showing survival benefits for ovarian cancer screening in women with pathogenic *BRCA* mutations. However, screening with concurrent transvaginal ultrasound and CA-125 is commonly used in mutation carriers who have not undergone bilateral salpingo-oophorectomy (BSO) and is suggested as an option in the current National Comprehensive Cancer Network (NCCN) guidelines [68].

Over the past two decades, risk-reducing surgeries for ovarian cancer arising in *BRCA1* and *BRCA2* mutation carriers have gradually strengthened the concept that most high-grade serous ovarian cancers arise in the fimbria of the fallopian tubes and go through a premalignant stage (identified by p53 immunostaining), an intraepithelial neoplasia stage (serous tubal intraepithelial carcinomas or STICs), and then the development of invasive carcinoma [69]. Occasionally the tubes and ovaries are totally fused within a neoplastic mass at the time of surgery, and often the cancer is already disseminated throughout the peritoneal cavity at diagnosis.

Given the limitations of screening tests and the poor outcomes associated with advanced high-grade serous ovarian cancers, the focus has been on preventive strategies for women at high risk for breast and ovarian cancer, including *BRCA* mutation carriers. Risk-reducing BSO is recommended for women with pathogenic *BRCA* mutations and who have completed childbearing, either by age 35–40 or individualised based on onset of ovarian cancer in family members, based on studies that have found that BSO significantly reduces both the risk of ovarian cancer (by 70%–80%) and all-cause mortality in *BRCA* mutation carriers [70, 71] and may also have a protective effect on breast cancer risk if completed prior to menopause [70, 72]. It is important to note that a continued, small risk of primary peritoneal cancer persists after BSO.

A major clinical issue is when to institute risk-reducing surgeries in premenopausal women who are not ready for BSO, either because they are contemplating childbearing or for other reasons. Since a majority of high-grade serous ovarian cancers may arise from the fallopian tubes, salpingectomy with delayed oophorectomy is an alternative potential strategy for these patients and is currently being studied in the women choosing surgical prevention clinical trial (NCT02760849).

Other preventive measures include the use of oral contraceptives for ovarian cancer risk reduction. A meta-analysis of six studies evaluating ovarian cancer risk in *BRCA* 1/2 mutation carriers and eight studies evaluating breast cancer risk in *BRCA* 1/2 mutation carriers found an inverse association between oral contraceptive use and ovarian cancer risk (odds ratio, 0.58; 95% confidence interval (CI), 0.46–0.73), but a non-statistically significant trend towards an increased breast cancer risk (odds ratio, 1.21; 95% CI, 0.93–1.58) [73]. Other data assessing oral contraceptive use and breast cancer risk in *BRCA* mutation carriers have been conflicting [74, 75]. Thus, women with *BRCA* mutations should be carefully counselled on the benefits and potential harms of oral contraceptives prior to using them as an alternative way to reduce the risk of ovarian cancer.

Even though deleterious mutations in both *BRCA1* and *BRCA2* result in defects in HR and predispose patients to develop breast and ovarian cancers, most breast cancers that develop in *BRCA1* mutation carriers are triple negative, whereas most breast cancers in *BRCA2* mutation carriers are hormone receptor-positive, HER2-negative. This difference affects patient counselling since preventative measures may have more or less of an impact depending on whether the patient is a carrier of *BRCA1* or *BRCA2*. For example, *BRCA2* carriers may have more of a reduction in breast cancer risk with risk-reducing salpingo-oophorectomy than *BRCA1* carriers based on a prospective multicentre cohort study [70] although another study found no significant reduction in risk of first breast cancer with BSO, even in *BRCA2* carriers.

### Treatment

The treatment of *BRCA*-mutated advanced ovarian cancer is distinguished from the general treatment of advanced ovarian cancer (without germline *BRCA* mutations), mainly through the inclusion of PARP inhibitors into management plans. A complete discussion of PARP inhibitors in ovarian cancer is beyond the scope of this review. We briefly summarise what is provided by Physician Data Query (PDQ) in www.cancer.gov.

Two PARPis are currently FDA-approved for the single-agent treatment of women with *BRCA* mutations and recurrent ovarian cancer: olaparib and rucaparib. Olaparib is approved for use in women with recurrent ovarian cancer and a germline *BRCA* mutation after three or more prior lines of treatment, and rucaparib is indicated for use in women with recurrent ovarian cancer and a known *BRCA* mutation (either somatic or germline) after two or more prior lines of treatment.

Phase 2 trials of olaparib and rucaparib demonstrated excellent objective response rates and progression-free survival, with better outcomes in patients with platinum-sensitive disease compared to patients with platinum-resistant disease [5, 76–78]. Olaparib also demonstrates activity in platinum-resistant ovarian cancer, as seen in a phase 2 basket trial of patients with germline *BRCA 1/2* mutations and recurrent cancer. In the cohort of 193 patients with platinum-resistant ovarian cancer, 31% achieved an objective response [79].

The other three PARPs, niraparib, veliparib and talazoparib, are also being evaluated in advanced ovarian cancer in monotherapy and combination therapy trials. Ongoing combination therapy trials are assessing PARPis with chemotherapy, immunotherapy and targeted agents, including PI3-kinase inhibitors, ATM inhibitors and angiogenesis inhibitors.

Three PARPis are currently FDA-approved for the *maintenance* treatment of patients with platinum-sensitive relapsed ovarian cancer who had a partial or complete response to platinum-based chemotherapy, regardless of *BRCA* status: niraparib, olaparib and rucaparib. Approval was based on randomised trials that demonstrated a progression-free survival benefit to PARP inhibition compared to placebo [80–82]. Subgroup analyses from these trials demonstrated a greater benefit for patients with germline *BRCA* mutations compared to patients without germline *BRCA* mutations [80]. Most recently, olaparib was approved in the first line maintenance setting for patients with advanced ovarian cancer and pathogenic *BRCA* mutations, following the results of a phase 3 randomised trial that showed that 2-year consolidation resulted in a significantly improved progression-free survival that was sustained after drug discontinuation, at a median follow-up of 36 months [83].

## Prevention and treatment of *BRCA*-mutated breast cancer: current status

### Prevention

Risk-reducing prophylactic bilateral mastectomy has been shown to decrease the incidence of breast cancer by more than 90% in women at risk of hereditary breast cancer [70]. The NCCN, therefore, recommends that prophylactic bilateral mastectomy be offered to *BRCA* carriers [68]. However, in contrast to ovarian cancer, effective screening tests (mammogram and MRI) for breast cancer exist and so the choice to undergo prophylactic bilateral mastectomy should be individualised to the patient and based on her personal preferences. As mentioned previously, risk-reducing BSO may reduce the risk of breast cancer in *BRCA* mutation carriers if completed prior to menopause, although the extent of breast cancer risk reduction has varied considerably between studies [70, 72].

Current non-surgical prevention strategies are mainly limited to chemoprevention with tamoxifen, a selective oestrogen receptor modulator. There is insufficient data addressing the preventive benefit of aromatase inhibitors or raloxifene for patients with *BRCA* mutations although several trials have shown that these drugs reduce the risk of breast cancer in the general population of higher risk women. The data supporting tamoxifen use in *BRCA* mutation carriers are also relatively limited; subset analyses of a National Surgical Adjuvant Breast and Bowel Project Breast Cancer Prevention trial (P-1 trial) demonstrated a reduction in breast cancer risk with tamoxifen use for patients with pathogenic *BRCA* mutations [84]. The preventive benefit of tamoxifen was mainly noted in *BRCA2* mutation carriers and not in patients with *BRCA1* mutations, likely because *BRCA2*-mutated breast cancer tends to be hormone receptor-positive. Even though tamoxifen may primarily reduce the risk of hormone receptor-positive breast cancer, prospective studies of mutation carriers with a diagnosis of breast cancer and who took tamoxifen showed a reduction in contralateral breast cancers, including a significant reduction in *BRCA1*-associated breast cancers, suggesting that tamoxifen may also reduce the risk for triple negative breast cancers [85, 86]. Overall, data supporting tamoxifen use in *BRCA* mutation carriers are more limited than chemoprevention data for the general population of high-risk women. Tamoxifen can be considered for women who decline prophylactic mastectomies, especially patients with *BRCA2* mutations, but patients should understand that risk-reducing prophylactic mastectomies are more effective in reducing breast cancer risk.

Other strategies for chemoprevention of both *BRCA*-associated breast and ovarian cancers are being actively evaluated in preclinical and clinical studies. As discussed previously, RANKL inhibition holds particular promise and is being evaluated in a pilot clinical trial (NCT03382574). Letrozole, recombinant human chorionic gonadotropin and lifestyle changes are also being evaluated as preventive measures for *BRCA*-associated breast cancer in clinical trials (NCT00673335, NCT03495609 and NCT02516540).

### Treatment

The treatment of patients with advanced breast cancer and deleterious *BRCA* mutations was not appreciably different from the treatment of patients with wild-type *BRCA* status and advanced breast cancer until studies indicated that patients with *BRCA* mutated-breast cancer were especially sensitive to treatment with platinum chemotherapy. The largest of these studies was the phase 3 Triple Negative Trial (TNT) trial, which randomised patients with advanced triple negative breast cancer to treatment with either carboplatin or docetaxel. In the unselected population, there was no significant difference in activity between the two therapies, but in patients with germline *BRCA* mutations, carboplatin demonstrated a significantly longer progression-free survival and overall response rate compared to docetaxel (overall response rate 68% versus 33%, respectively, *p* = 0.01) [87].

PARP inhibitors were then evaluated in advanced *BRCA*-mutated breast cancer, following compelling results from ovarian cancer clinical trials. OlympiAD was a randomised phase 3 trial, in which olaparib monotherapy was compared with standard physician’s choice chemotherapy (capecitabine, vinorelbine or eribulin) in patients with germline *BRCA* mutations and metastatic HER2-negative breast cancer. The progression-free survival was 7.0 months in the olaparib group and 4.2 months in the physician’s choice chemotherapy group (hazard ratio 0.58, *p* < 0.001) [88]. The larger phase 3 EMBRACA trial evaluated talazoparib (a PARPi with potent PARP trapping ability) versus physician’s choice chemotherapy in a similar patient population with HER2-negative advanced breast cancer and deleterious germline *BRCA 1/2* mutations, and similarly found a longer progression-free survival in the talazoparib arm with acceptable toxicity (8.6 months versus 5.6 months, *p* < 0.0001) [89]. There was no significant difference in overall survival in the OlympiAD trial (the trial was not powered to detect a difference), and overall survival results are not yet mature for the EMBRACA trial. In January 2018, following the results from OlympiAD, the FDA approved olaparib for the treatment of patients with deleterious or suspected deleterious germline *BRCA*-mutated, HER2-negative metastatic breast cancer and who had been previously treated with chemotherapy either in the neoadjuvant, adjuvant or metastatic setting. Talazoparib subsequently gained FDA approval in October 2018 for patients with deleterious or suspected deleterious germline *BRCA*-mutated, HER2-negative locally advanced or metastatic breast cancer.

The unresolved question from these trials is how to fit PARP inhibitors into the treatment scheme for patients with germline *BRCA* mutations and advanced breast cancer, and specifically, how to sequence platinum chemotherapy with PARP inhibitors. Subgroup analyses from OlympiAD and EMBRACA show that in patients who had previously received platinum chemotherapy, response rates to the PARP inhibitors were lower than other subgroups. The phase 2 ABRAZO trial evaluated talazoparib in patients with advanced breast cancer and germline *BRCA* mutations and demonstrated a progression-free survival of 4 months for patients who had progressed at least 8 weeks after the last dose of platinum chemotherapy. In sum, although the response to PARP inhibition may be lower in patients previously treated with platinum chemotherapy compared to platinum-naïve patients, PARP inhibitors still demonstrated efficacy in patients who had responded to platinum chemotherapy in the metastatic setting or had relapsed at least a few months after treatment.

An alternate strategy would be to treat early in the metastatic disease course with PARP inhibition and then follow with platinum chemotherapy at progression. A phase 2 trial of patients with germline *BRCA 1/2*-mutated metastatic breast cancer assessed single-agent veliparib, another PARPi, followed by veliparib plus carboplatin at disease progression. The post-progression treatment with veliparib and carboplatin at the maximum tolerated doses (150 mg twice per day and area under the curve (AUC) of 5, respectively) yielded minimal benefit; only one patient out of 30 had a response [90]. Overall, the optimum sequence of therapy in patients with germline *BRCA*-mutated metastatic breast cancer still needs to be determined.

An ongoing randomised, prospective, phase 2 clinical trial is comparing cisplatin with or without veliparib in patients with metastatic triple negative and/or *BRCA*-mutated breast cancer (NCT02595905). A hypothesis is that combination therapy, if tolerated, could be more effective than sequential therapy since it may attenuate the development of cross-resistance. Overlap between the mechanisms of platinum and PARP inhibitor resistance is being explored. However, a recurring issue in trials combining PARP inhibitors with chemotherapy is the dose-limiting toxicity, particularly myelosuppression. Most of the combination trials with chemotherapy and PARP inhibitors have dealt with the toxicities by prioritising the chemotherapeutic agent and using lower doses of the PARP inhibitor, such as in the phase 3 BrighTNess trial, which showed no improvement in pathologic complete response rate with the addition of veliparib to neoadjuvant carboplatin and paclitaxel in triple negative breast cancer [91]. Managing these toxicities and finding effective doses of both the PARP inhibitor and the chemotherapy agent remain significant challenges for chemotherapy and PARP inhibitor combination trials.

In terms of the treatment of *BRCA*-mutated early stage breast cancer, neoadjuvant or adjuvant platinum-based chemotherapy is commonly used, as supported by a few studies showing that platinum-based neoadjuvant chemotherapy leads to improved rates of pathological complete response [92, 93]. Of course, platinum-based chemotherapy is not appropriate for all patients (elderly, poor performance status and low-risk early-stage cancers) given the additional toxicity. These studies are also not definitive since they did not show improvements in disease-free and overall survival, were not restricted to patients with *BRCA* mutations or were not large randomised studies. However, when taken with the compelling results from the TNT trial, platinum-based neoadjuvant or adjuvant chemotherapy is favoured for *BRCA*-mutated patients. PARP inhibitors have not yet shown consistent activity in the neoadjuvant setting [91] and are not presently indicated in the neoadjuvant or adjuvant treatment of *BRCA* mutated-early stage breast cancer. However, a few PARPis are currently in phase 2–3 clinical trials assessing adjuvant or neoadjuvant therapy in women with HER2-negative breast cancer and germline *BRCA 1/2* mutations.

## Emerging therapeutic concepts in targeting HRD

This growing understanding of how *BRCA* mutations drive carcinogenesis has led to new concepts in the prevention, diagnosis and treatment of breast, ovarian and related gynaecologic cancers.

### PARP inhibitors and PARP inhibitor (PARPi) resistance

The PARP family of enzymes functions by transferring ADP-ribose from nicotinamide adenine dinucleotide (NAD)+ to other proteins and is involved in a number of DNA repair processes. PARPis have demonstrated clinical activity in *BRCA* mutated breast and ovarian cancer and are thought to induce synthetic lethality in these tumours through the interaction between the HRD from BRCA deficiency and four PARPi-associated effects: defective base excision repair, PARP trapping on damaged DNA (PARP1 is unable to disassociate from DNA leading to obstructed replication forks), defective BRCA1 recruitment and activation of NHEJ [94].

Despite the demonstrated efficacy of PARP inhibition in *BRCA*-mutated breast and ovarian cancers, most patients eventually develop resistance, as evidenced by a median progression-free survival of less than 1 year in the major PARPi clinical trials. Several mechanisms of PARPi resistance have been described and include 1) increased PARPi drug efflux; 2) reversion mutations in *BRCA* or other mutated genes that restore protein function and thus restore HR; 3) increased NAD+ synthesis which mitigates PARP trapping; 4) loss of 53BP1 and REV7 and 5) replication fork stabilisation [95]. The development of reversion mutations that restore HR may be the most well-known mechanism of PARPi resistance [96]. Loss of p53-binding protein 1 (53BP1) is another significant PARPi resistance mechanism. 53BP1 acts together with RIF1 and inhibits the end resection step of HR, antagonising the function of BRCA1. Correspondingly, loss of 53BP1 has been shown to restore HR, even in cells with BRCA deficiency [29, 97].

Strategies to circumvent PARP inhibitor resistance are a major focus of preclinical research and clinical trials. One general strategy is an attempt at augmenting synthetic lethality by targeting other DNA damage repair mechanisms in combination with PARP inhibition, such as the ATR or ATM pathways. A few ongoing clinical trials are using this approach in both advanced breast and ovarian cancers (clinicaltrials.gov).

Recent studies have demonstrated that tumours deficient in HR up-regulate the MMEJ DNA repair pathway as a survival mechanism [30]. *In vitro* studies using HR-deficient ovarian cancer cell lines found that knockdown of MMEJ reduced the survival of the cells after exposure to both PARP and ATM inhibition [98]. Thus, the increase in the activity of the MMEJ pathway could be a way for tumours to maintain viability while receiving PARP inhibition, leading to PARPi resistance. This concept needs to be validated through further preclinical studies.

### Immunotherapy

Recently, deficiencies in DNA repair processes have emerged as predictive biomarkers for response to immune checkpoint blockade. In ovarian cancer, tumour specimens from patients with high-grade serous ovarian carcinoma and BRCA1 or BRCA2 loss (through germline or somatic mutations, or *BRCA* methylation) had significantly increased immune cell infiltrates [99] and overexpression of PD-L1 [100] compared to high-grade serous ovarian cancer specimens without *BRCA* mutations. Recently, the preliminary results from TOPACIO/Keynote-162 were presented at the American Society of Clinical Oncology (ASCO) annual meeting in June 2018. This is a phase 2 trial evaluating the combination of niraparib, a PARPi, and pembrolizumab in platinum-resistant ovarian cancer. The primary endpoint was overall response rate, which was 25% in all patients and 45% in patients with *BRCA* mutations [101]. Follow-up is ongoing.

In breast cancer, a preclinical study using breast cancer cell lines showed that PARP inhibition up-regulated PD-L1 expression, and that combination treatment with PARP inhibition and anti-PD-L1 therapy was significantly more efficacious than either treatment alone [102]. The TOPACIO/Keynote-162 study in triple negative breast cancer was also presented at the recent ASCO 2018 annual meeting. This phase 2 study evaluated niraparib plus pembrolizumab in patients with metastatic triple negative breast cancer, of which 22% had deleterious *BRCA* mutations and 41% had received platinum chemotherapy in the metastatic setting. The overall response rate for all patients was 29% and was significantly higher in the patients with *BRCA* mutations [103]. Follow-up for this study is ongoing.

Teo et al. [104] evaluated 60 patients with advanced urothelial cancer from prospective clinical trials featuring nivolumab or atezolizumab and found that 25% of these patients had likely deleterious DNA damage repair mutations. These patients had a markedly better overall response rate compared to patients without DNA damage repair mutations (80% versus 19%, *p* < 0.001).

Taken together, immune checkpoint inhibition appears to have promising activity in tumours with HRD, including breast and ovarian cancers. Combination therapies with immune checkpoint inhibitors and agents targeting DNA damage repair are particularly intriguing since the neoantigen load may be further increased with drugs targeting DNA repair (such as PARPis) leading to a more immunogenic tumour. Multiple ongoing or planned clinical trials are evaluating immune checkpoint inhibitors alone or in combination with other drugs in breast and ovarian cancers with HRD (see Table 2 below).

## Conclusions

The cloning and discovery of the *BRCA 1/2* genes over two decades ago drove a body of research on the biology of BRCAness that continues to expand today. Advances in understanding the interplay of DNA repair processes and the interaction between the DNA damage response and other host factors, such as the immune system and hormone regulation, are being translated into clinical trials in ovarian and breast cancer. Our growing knowledge of HRD is being developed into biomarkers for treatment response, and several assays for HRD are undergoing clinical validation. The role of PARP inhibition is also expanding. PARPis are being evaluated in new and earlier settings, such as first-line maintenance therapy and platinum-resistant disease in ovarian cancer and in neoadjuvant therapy in breast cancer. Combination therapies of PARPis with other agents are being evaluated in breast and ovarian cancer clinical trials, as a way to circumvent PARP inhibitor resistance, augment synthetic lethality and increase response. Increasingly, we recognise the heterogeneity of patients with *BRCA* mutations and breast or ovarian cancer and appreciate that many different driver processes may be at play in tumorigenesis. Thus, the future of BRCA-like breast and ovarian cancer treatment, like other cancers, may feature a designer therapeutic approach, in which we query which pathways are active in a specific tumour and target those.

## Funding

The authors did not receive any funding for the preparation of this article.

## Conflicts of interest

The authors declare no conflict of interest.

## Figures and Tables

**Table 1.** Tracking our progress in BRCA genes: symposia on prevention and treatment.

Conference (sponsor)	Location	Date	Highlights
Screening and prevention in women’s cancer I (LCF)	USC/Norris Los Angeles	26 April 2003	**Prophylactic surgery: results, issues** (L Hartmann- Mayo Clinic) **MRI for early detection** (M Schnall—University of Pennsylvania)
II (LCF)	NYU	15–16 April 2005	**MRI versus mammogram/US in *BRCA*mu** (E Warner—University of Toronto); ***BRCA*1/2 genome caretakers** (J Boyd—Memorial Sloan-Kettering Cancer Center)
III (LCF)	NYU	4–5 May 2006	**Expanding biomarkers fall short** (N Urban—Fred Hutchinson Cancer Research Center); **non-ovarian origin of serous ‘ovarian’ cancer! **(R Drapkin, L Dubeau—University of Southern California)
1st Joint HBOC meeting Bari NCI and NYU (NCI)	Aula Magna Bari	10–12 September 2009	***BRCA* susceptibility genes; PALB2** (D Silver—Dana-Farber Cancer Institute; I Catucci—Istituto Europeo di Oncologia); **DNA repair: a therapeutic target** (J Jonkers—The Netherlands Cancer Institute; H Calvert—Newcastle University)
2nd (NCI) ‘lessening the burden’	NYU	14–17 September 2011	**Keynote: Learning from hereditary cancers** (H Lynch—Creighton University);** DNA repair & genetic instability** (J Boyd—Fox Chase Cancer Center; R Parsons—Columbia University)
5th McGill BRCA Symposium (Eisman, Foulkes)	Montreal	23–25 April 2014	**Hierarchy of prevalence and cancer susceptibility genes;** ***BRCA 1* functions** (D Livingston—Dana-Farber Cancer Institute)
3rd Joint HBOC Bari/NYU	Bari Town Hall		**Keynote: Prevention via E/P modulation** (M Pike—Memorial Sloan Kettering Cancer Center); **haploinsufficiency→2nd cancers **(S Pathania—Dana-Farber Cancer Institute)
6th McGill BRCA Symposium	Montreal	10–13 May 2016	**POLQ and potential therapeutic implication in BRCA-related cancers** *(*A Sfeir—NYU*);* **prevention-salpingectomy**
7th	Montreal	8–11 May 2018	**PARPi/platinum resistance** (D Silver—Thomas Jefferson University; N Turner—The Royal Marsden Hospital and The Institute of Cancer Research); **RANK and osteoprotegerin decoy receptor: BC risk **(J Katsopoulos—University of Toronto)

Abbreviations: LCF = Lynne Cohen Foundation; USC = University of Southern California; NYU = New York University; US = ultrasound; *BRCA*mu = *BRCA* mutation carriers; HBOC = hereditary breast and ovarian cancer; NCI = National Cancer Institute; E/P = oestrogen/progesterone; PolQ = gene encoding for the DNA polymerase theta; PARPi = PARP inhibitor; BC = breast cancer

**Table 2.** Ongoing clinical trials with immunotherapy in BRCA-like breast and ovarian cancer.

Study	Phase	Disease and stage	Metastatic line of treatment	BRCA and/or HRD status requirement	Intervention
NCT03025035	2	Advanced BC	≥2	gBRCAm	Single-agent *pembrolizumab*
NCT02393794	1/2	Metastatic TNBC, or locally recurrent or metastatic gBRCAm BC regardless of subtype	At least one line of therapy in advanced or adjuvant setting	A subset of patients have gBRCAm and the rest have metastatic TNBC and wild-type BRCA	Cisplatin, romidepsin, *nivolumab*
NCT01898117	2b	Metastatic or locally advanced TNBC	1	Trial validates a BRCA-like assay in predicting response to treatment	Carboplatin-cyclophosphamide versus paclitaxel with or without atezolizumab
NCT03101280	1b	1) Dose-finding phase: advanced OC or endometrial cancer 2) Dose-expansion phase: platinum-sensitive OC or advanced TNBC	≥2	The dose-expansion phase is limited to patients with *BRCA* mutations (germline and somatic) or BRCA-like molecular signature (LOH)	Rucaparib and *atezolizumab*
NCT02849496	2	Unresectable stage III TNBC or metastatic TNBC	Any	*BRCA* 1/2 mutation (germline or somatic)	Olaparib with or without *atezolizumab*
NCT03414684	2	Metastatic TNBC	1 or 2	Subset of patients have *BRCA* mutations	Carboplatin with or without *nivolumab*
NCT03428802	2	Advanced solid tumour	Any	Arm 1: DNA polymerase epsilon (POLE) and POLD1 mutation Arm 2: *BRCA* 1/2 mutation (germline or somatic)	Single-agent *pembrolizumab*
NCT03206203	2	Metastatic TNBC	1 or 2	Subset of patients have *BRCA* mutations	Carboplatin with or without *atezolizumab*
NCT03330405	1b/2	Incurable locally advanced or metastatic solid tumour	Any	*BRCA* 1/2 or ATM mutation	Talazoparib and *avelumab*
NCT02734004	1/2	Platinum-sensitive relapsed small cell lung cancer; gBRCAm HER2-negative metastatic BC; gBRCAm OC; metastatic or relapsed gastric cancer (adenocarcinoma); or gBRCAm-negative OC	Any	See disease and stage	*Durvalumab* and olaparib; for patients with gBRCAm-negative OC, a subset receives *durvalumab*, olaparib and bevacizumab
NCT02953457	1/2	Platinum-sensitive or platinum-resistant recurrent or persistent or refractory ovarian, fallopian tube, or primary peritoneal carcinoma	Any line of treatment for recurrent/persistent/refractory disease	*BRCA* 1/2 mutation (germline or somatic), or mutation in another HR gene, or evidence of HRD on LOH score	Olaparib, *durvalumab* and tremelimumab
NCT03100006	1b/2a	Advanced epithelial OC	≥3 (one of the prior treatment lines includes platinum and taxane)	Subset of patients have *BRCA* mutations	Oregovomab vaccination with *nivolumab*
NCT03394885 (AdORN)	1b	Stage III or IV epithelial OC, fallopian tube or primary peritoneal carcinoma	1 (previously untreated patients)	Subset of patients have *BRCA* mutations	Neoadjuvant and adjuvant *atezolizumab*, carboplatin, paclitaxel with interval cytoreductive surgery

Abbreviations: HRD = homologous recombination deficiency; BC = breast cancer; g*BRCA*m = germline *BRCA 1/2* mutated; TNBC = triple negative breast cancer; OC = ovarian cancer; LOH = loss of heterozygosity; HR = homologous recombination
